# Keeping Track of the Genealogy of Heterozygotes Using Epigenetic Reference Codes and Breeding Tables

**DOI:** 10.3389/fnbeh.2021.781235

**Published:** 2022-02-11

**Authors:** Anna Sara Liberati, Barbara Calcaprina, Walter Adriani

**Affiliations:** ^1^Faculty of Psychology, Università Telematica Internazionale “Uninettuno,” Rome, Italy; ^2^Center for Behavioral Sciences and Mental Health, Istituto Superiore di Sanità, Rome, Italy

**Keywords:** heterozygote, taxonomy, genealogy, dopamine, breeding strategy, parent of origin effect

## Abstract

Studying neurobehavioral consequences of the hypofunctional dopamine transporter (DAT) across several generations entails the need to monitor allelic transmission to offspring, taking into account both maternal and paternal inheritance. Since each type of heterozygote expresses differential phenotypes, based on lineage of inheritance for wild and mutated alleles (from male or female ancestors), it is important to track transgenerational epigenetic effects. We deemed it essential to assign specific abbreviations identifying their characteristics. Therefore, we devised a Mendelian-inspired table to keep track of these. Starting from two progenitors (WT and KO) we named resulting heterozygous progenies MAT and PAT to differentiate them based on inheritance of the wild allele (from the mother or father). Tracing subsequent generations, similar logic has been followed: if coupling HET dams with KO males, initials “M” [(grand)maternal] and “P” [(grand)paternal] are kept, but “AT” is turned into “IX” (MIX and PIX), while if breeding HETs with WTs, “M” is changed to “W” resembling an upside down “M” and “P” to “S” for “sperm” (WAT and SOT). To underline the development within “hyperdopaminergic-uterus” a central letter “U” is added (MUX, PUX, and QULL), while a Greek initial (μAT, μIX, and νIX) underlines the uterine-worsened origin of the allele. In HET × HET breeding (GIX and DIX), the mutated allele can be inherited from both sides of the genealogical line. However, when the mother is MAT, wild and mutated alleles encounter for the first time, causing putative anomalies in the progeny. Replacing dam with a second-generation female (MIX and MUX) may mitigate epigenetic effects on third-generation offspring; therefore suffixes (“-f,” “-fu,” “-ϕ,” and “-ϕu”) emphasize that subsequent-generation dams imply that the alleles already encountered in HET (rather than WT) grand-dams.

## Foreword

Accurate genetic monitoring of laboratory colonies of genetically modified animal models (GA) should be an essential component of laboratory work and observations (Benavides et al., 2020). To keep this simple, classical breeding may exploit heterozygous × heterozygous mating. However, a need may arise to cross different phenotypes in order to obtain specific GA lines. In such cases, a study on their genetic, phenotypic, and behavioral characteristics with an aim to discriminate paternal and/or maternal effects in offspring will obviously generate complexity and confusion. Therefore, it is essential to adequately monitor all relevant information related to various epigenetic features that could arise from: quality of maternal care, transgenerational transmission of the mutated allele by maternal, paternal or both lineages, up to fetal development within a hyperdopaminergic uterus (and consequent sequelae), and so on.

Such data should always include the correct name of the strain, detailed description of its characteristics, genetic/epi-genetic background, observed phenotypic and behavioral variations, etc. For this purpose, some standardized cataloging protocols and specific guidelines are now available, such as the technical data sheets proposed by the Federation of European Laboratory Animal Science Association (Bonaparte et al., 2013). However, it could also be very useful to have simple genealogical tables at hand, allowing to obtain adequate information at “a glance.”

In our recent research work, we dealt with the study and observation on dopamine transporter (DAT) hypofunctional heterozygous rats. A classical type of heterozygous offspring was obtained by breeding parents that were both heterozygous for the DAT gene, and this led to a “mixed” offspring with Mendelian proportion of all genotypes, initially named, for this reason, MIX-HET. Lately, it was decided to focus the observation specifically on heterozygous pups, obtained by breeding a knocked-out (KO) subject for the DAT gene and a wild-type (WT) one (Adinolfi et al., 2019; Carbone et al., 2020). The breeding was carried out following both of two possible combinations: the first one in which the males were KO and the females were WT (from which we obtained a “maternal line” offspring, thus named MAT-HET) (Zelli et al., 2021), and the other in which, instead, the males were WT and the females were KO (from which, therefore, a “paternal line” offspring was obtained, consequently named PAT-HET). At that point, we noted many behavioral differences between the two genealogical lines (some of which are described in section “Sociality and Spontaneous Circadian Rhythms of Locomotor Activity in MAT-HETs”), which were initially attributed to the influence of a discrepant maternal care behavior, since the presence of the hypofunctional DAT allele in HET mothers is known to play a relevant role in caring behavior (Mariano et al., 2020; Brancato et al., 2021). Specifically, a “depressed-like” phenotype may actually depend on the altered care given to offspring by a heterozygous dam (Mariano et al., 2020). However, the experiment intended to verify this relationship (Oggiano et al., 2022) rather told us that the “asset” (i.e., inheriting the allele from the mother or father) was far more important than maternal care (see section “Sociality and Spontaneous Circadian Rhythms of Locomotor Activity in MAT-HETs”). Thus, we decided to investigate how and in which measure allelic distribution (therefore the “asset”) had influence over the behavior expressed by DAT-HET strains throughout subsequent generations. Therefore, we carried on our colony by breeding together, in various combinations, the original maternal and paternal lines in order to evaluate the epigenetic effects of the mutated allele owing to its different distributions in the genealogical tree, thus obtaining more generations and types of heterozygous rats, each with its own specific characteristics. Hence, the need to develop a practical and easily manageable strategy arose, in order to keep constant and instant track of the nominal acronyms and pedigree origins. Although, of course, there are only three possible genotypes (WT, HET, and KO), many different epi-genotypes may result from their breeding, precisely by virtue of the epigenetic effects that we ascertained. In other words, rats with the same genotype may express themselves differently based on the maternal or paternal derivation of the mutated allele. For this purpose, following the “Ockham’s razor” principle (which asserts that the most practical solution is often the most obvious and banal one), we decided to exploit a simple cross-graph genealogical table. In this way, any research group could have the possibility, with a quick glance, to get an immediate “resume” of the genealogical line, the nominative abbreviation and allelic distribution (“asset”) of the gene throughout different generations, avoiding any confusion.

## Sociality and Spontaneous Circadian Rhythms of Locomotor Activity in MAT-HETs

Human individuals shape dynamic and complex societies that are based on continuous reciprocal interactions. According to the “social brain hypothesis” (Dunbar Robin, 1998), sections of the human neocortex have evolved to enhance survival in complex communities and process social knowledge. The importance of social experiences in human activity is also evident in many psychological disorders, where impairments of social interaction are an important part of the diagnosis (e.g., autism spectrum disorder, schizophrenia, social phobia, etc.). In fact, as demonstrated by several pharmacological studies, many neurotransmitters, such as oxytocin, dopamine, noradrenaline, and β-endorphin, are recruited in social behavior (Pearce et al., 2017). As a result, social interaction has recently received further attention in cognitive neuroscience as well as in other fields of psychology and psychotherapy.

Dopamine (DA) is a neurotransmitter that plays a key role especially in action and motivation; it is commonly recognized as being associated with pleasure as well as with a number of other functions, such as movement, memory, and attention. It is released during appetitive and/or consuming phases when we eat, have sex, engage in physical activity, and when we socially interact. It “rewards” us for positive outcomes arising from our action and choices; therefore, it motivates us to replicate such behaviors until they become habits or even addictions. Emotive eating, sex, and drug abuse are, in fact, often linked to the release of DA in the nucleus accumbens and prefrontal cortex. In the mammalian brain, there are four main dopamine pathways: mesolimbic, mesocortical, nigrostrial, and tuberoinfundibular. The mesocortical, mesolimbic, and nigrostriatal pathways constitute our “reward system” and have been shown to be defective in the majority of cases of addiction. The nigrostriatal pathway is involved in motor planning, while the tuberoinfundibular pathway inhibits prolactin release. Dopamine therefore has a variety of effects on motor activity (Centonze et al., 2003). Furthermore, the dopamine transporter (DAT) protein enables the uptake of dopamine into presynaptic terminals, thus interrupting its function (Giros et al., 1996).

Failure of the reuptake by DAT can, therefore, imply a variety of consequences for the proper function of the dopaminergic system. In rats, silencing the gene encoding the dopamine transporter (DAT SLC6A3) triggers odd behavioral patterns. By means of biotechnologies, a stop codon has been inserted in the reading frame of the gene; hence the DAT protein gets truncated after less than 70 amino acids. Therefore, the truncated DAT rat, by silencing the activity of SLC6A3, represents an interesting new animal model for research. Fully truncated DAT (KO) rats, for example, show a typical behavioral profile that involves hyperactivity, working memory deficiency, higher proclivity to develop stereotyped behaviors, and a variety of other symptoms attributable to hyperdopaminergy (Cinque et al., 2018; Leo et al., 2018; Adinolfi et al., 2019; Carbone et al., 2020). The truncated-DAT allele also results in different sociability profiles.

On the other hand, heterozygous (DAT-HET) rats exhibit asocial tendencies, which are caused by DAT haploinsufficiency (Adinolfi et al., 2019). Another result of dopaminergic system dysfunction is disruption of circadian rhythms: KO rats typically show difficulties in falling asleep; therefore, their resting phase is shortened. It is known that circadian rhythm disturbance can be the cause of a number of psychological and psychiatric issues (Stranges et al., 2012). Epigenetic influences, such as those due to inadequate maternal cares, may affect DAT expression, thus resulting in circadian hypofunctionality (Mariano et al., 2020) and, consequently, behavioral anomalies. In general, maternal inherited gene expression appears to have been favored by evolution, especially during brain maturation (Gregg et al., 2010a,b). However, despite the fact that fully blocking DA reuptake induces hyperactivity and stereotypies, in heterozygous (HET) subjects, the phenotypic effects of inheriting the truncated allele from the mother or from the father are currently unknown. Thus, observing the outcome of the transmission of the mutated allele in different generations of heterozygous offspring obtained from different combinations and different assets (maternal and/or paternal inheritance) could allow us to better understand these effects.

### Results From Atypical Breeding

Heterozygous subjects are classically obtained by HET/HET breeding in normal colony context. Since we observed reduced care by HET mothers during lactation, we decided to replace them with WT mothers. Thus, a heterozygous group was obtained from KO male rats and WT dams. Hence, this group was named MAT-HET (“maternal-heterozygotes”) (Carbone et al., 2020), since the functional DAT allele in the offspring was, always in this case, inherited from the mother. The offspring, of course, likely received a full repertoire of maternal care (see Mariano et al., 2020: “HETs with WT dam” corresponding to present MAT-HET).

Classical heterozygous born from HET/HET breeding are presently termed HET^2^. During the last year we realized that, in order to properly compare offspring differing only for maternal care, it was necessary to plan KO fathers for both groups. Therefore, starting from the fifth generation in the colony, a group of heterozygous males, which we named MIX-HET, was obtained by breeding KO male and DAT-heterozygous female subjects. Both MIX and MAT are heterozygous; therefore, variability is undoubtedly attributable to epigenetic effect: hence, we decided to refer to the two groups as “epi-genotypes.” Preliminary studies on MAT-HET rats (Carbone et al., 2020; Zelli et al., 2021) outlined some differences compared to HET^2^ rats (formerly termed MIX-HET as explained above). Increased overall locomotor activity was shown in MAT-HETs: they also seemed to be more active compared to HET^2^ rats during the first hour of lighting in the facility room (resting time) suggesting difficulties in falling asleep. During the Porsolt Forced Swim test, the MAT-HETs exhibited a highly unstable behavioral profile, in which passive floating and active escape behaviors frequently alternated one another. If transferred from animals to man, these observations could be translated in the alternation between states of despair and struggle due to altered emotional state.

Also, a social-preference test was performed using a two-chamber apparatus. The focal subject (either a MAT or a HET^2^ male) was let free to explore both chambers through the doorway and to meet, separately from each other, two caged individuals (termed *stimuli*). Focal and stimulus rats were separated only by a metal grid, whose 1-cm wide brackets were spaced 1 cm apart, leaving enough space to interact both visually and through olfactory signals. The social preference test was split into two sessions, either of which hosting different combinations of stimuli in both rooms. As described in one of our most recent articles (Brancato et al., 2021), it was observed that focal MAT-HET male rats spent more time in the chamber in which a female stimulus in estrus was caged than in the other room, where a female stimulus rat was, conversely, not in estrous. Focal HET^2^ males, on the other hand, tended to spend the same amount of time in either rooms, almost completely ignoring their occupants regardless of whether they were in estrus or not, thus denoting abnormal sexual drive.

As far as social interaction is concerned, the DAT heterozygous rats could be more informative than the full truncated (KO) DAT ones (Adinolfi et al., 2019; Sanna et al., 2020). In reality, when compared to WT and DAT-KO rats, classical HET^2^ rats show a strong asocial behavior and reciprocal lack of interest. DAT-HETs have only one functional allele for the DAT gene due to their heterozygous status. As a result, this particular allele appears likely more susceptible to epigenetic influences. Thus, we hypothesize that HET offspring may be composed of various epi-genotypes that would vary based on important factors such as the following: (1) inheriting the wild allele from mother or father; (2) whether they were cared for by a WT or a HET dam, since we observed that HET mothers are less inclined to provide appropriate maternal care. Furthermore, because of the atypical KO × WT breeding, MAT-HET rats are more likely to inherit unexplained DNA methylation and histone acetylation patterns from their KO fathers. Therefore, we run an experiment in order to dissect the two former points and try to avoid the latter (Oggiano et al., 2022).

For parental origin independently from foster dam (between factor 1) there were two conditions termed “assets”: (1) the “sick” maternal gene, also called “asset P” (born from WT father × HET mother) and (2) the “sick” paternal gene or “asset M” (born from HET father × WT mother). There were also two conditions in postnatal caring independent from asset (between factor 2) namely maternal care from a female WT rat versus from a female HET rat. We believed that dam care could be the main factor; therefore, the caregiver was never the natural mother but another female from the same colony, making the offspring comparable to the MAT and MIX offspring, respectively (Carbone et al., 2020). However, the parental origin of the truncated-DAT allele had a major effect, while the type of caregiving had none. Comparisons revealed significant differences in spontaneous locomotor activity between “sick”-maternal and “sick”-paternal gene subjects in three specific bins of the daily profile. As such, MAT and MIX codes are misleading. Indeed, in reconsidering our latest article (Oggiano et al., 2022), focal subjects were renamed here WAT (offspring of a WT dam and a MAT father) and SOT (offspring of a MAT female and a WT male) epi-genotype, to track pup’s parental origin.

Asset M subjects are similar to MATs, but they have WT siblings; therefore, we hereby propose to rename them “WAT.” Conversely, asset P subjects are completely inverted in comparison with the actual strategy for obtaining MIX rats (MAT dam × KO father). Indeed, by switching the father from a KO to a WT, we actually reversed the asset of chromosomes obtaining a new epi-genotype. Since the wild allele is inherited through the sperm, we propose to highlight this “spermatic origin” by adopting the abbreviation “SOT.” In the SOT epi-genotypes, increase in exploratory behaviors toward environment (Casarrubea et al., 2019) suggests a social disinterest in the same epi-genotype asset. However, disinterest seems to dissolve when involving rats that have opposite asset for parental origins.

### Discussion

The MAT-HET rats show an “almost bipolar-like” profile (Carbone et al., 2020) ranging from no interest for a HET male to great excitation and approach toward a receptive female. In fact, not only they are more socially attractive than the HET^2^ stimulus, they also appear much more euphoric than the hyposexual HET^2^ rats when they come into contact with a female (Brancato et al., 2021).

The hippocampus and hypothalamus of the HET rats show higher levels of norepinephrine, (NA) in response to the social stimulus (Adinolfi et al., 2019). In the HET rats, the immune-fluorescent experiment revealed a major change in noradrenergic transmission to the hippocampus and hypothalamus: compared to the WT and MIX-HET rats, the MAT-HET offspring showed large increase in NET immune-fluorescence in the hypothalamus and hippocampus (Brancato et al., 2021). Despite a thorough comparison of the hippocampal circuits and mechanisms underlying the diverse spectrum of social interactions has not yet been reported, previous research has shown that the hippocampal CA2 and CA3 regions are involved in social processing. Indeed, genetic defect in CA2 impairs social recognition, and CA3 pyramidal cell plasticity and transmission contribute to the encoding of social stimuli (Hitti and Siegelbaum, 2014; Chiang et al., 2018). Our results also indicate that altered noradrenergic transmission could play a role in the broad range of social changes observed in the HET rats.

These findings show that subjects with one truncated DAT allele have selectively different behavioral profiles when it comes to the maternal or paternal origin of the “sick” gene, but that there is no evidence of modulatory effect by postnatal caring. Compared to the wild ones, such alteration of the dopaminergic system makes individuals hyperactive (Leo et al., 2018; Carbone et al., 2020; Zelli et al., 2021). However, there are significant variations in the circadian rhythm of the studied epi-genotypes among subjects with a maternally or paternally inherited truncated DAT allele (Oggiano et al., 2022). Out of the whole cycle, two specific periods are affected. At the start of the resting period, subjects with “sick” maternal gene displayed substantially higher spontaneous locomotor activity than those with “sick” paternal gene, which could indicate difficulties in falling asleep. In brief, these results highlight the importance of the expression of the maternally inherited copy of the DAT gene in regulating the sleep-wake cycle (Dzirasa et al., 2006) through the dopaminergic system. It seems likely that endogenous factors (e.g., hormone levels) (Perera and Herbstman, 2011) may determine the preference for expression of the maternal DAT gene during uterine life.

Muglia et al. (2002) found that maternal meiosis is a major factor in evaluating a parent-of-origin effect on D4 dopaminergic receptors. A further suggestion is that epigenetic alteration of the dopaminergic system can determine social preferences (Oggiano et al., 2022). For what concerns the SOT epi-genotypes, data suggest disinterest in the same-asset epi-genotype; however, it seems that particularly WAT rats tend to avoid SOT subjects fostered to and cared from a WT dam. The better dams (or at least those that had regular WT-like care) paradoxically worsen the phenotype; thus a “sick” maternal allele is not reverted by enhancing the quality of care. Thus, high concentration of NA in the brain regions responsible for stress control could account for the socially abnormal behavior of MAT individuals, which are heterozygous for DAT (truncated DAT allele inherited from the father) and raised by a WT mother, as opposed to MIX subjects, which are heterozygous as well (but cared from a heterozygous DAT-HET female). It is possible to hypothesize concomitant NA hyperfunction and, vice versa, hypofunctionality of the DAT gene using this framework.

## Criteria and Genesis of Nominative Abbreviations

In order to realize, in practice, the genealogical table, we took into account the type of specimens considered to be the most significantly related to our studies. Therefore, not all combinations are present; rather, we chose those more likely to also describe phenotypes. We assigned an abbreviation for the already existing lines as well as for possibilities of future crossbreeding between them. We followed the maternal and paternal distributions of the mutated and wild alleles. For this reason, we decided to build an “8 × 8” table (Table 1A), which included male and female specimens of the following types:

**TABLE 1A:** Epigenetic acronyms for the progeny.

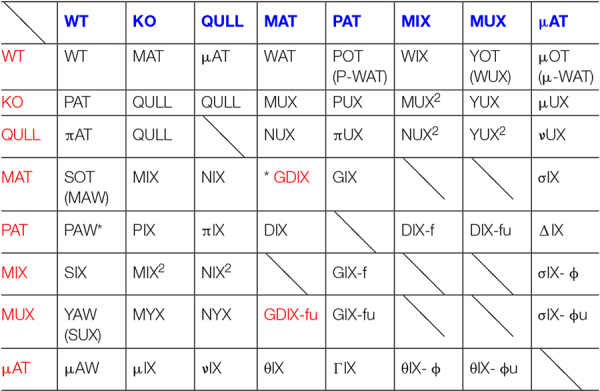

*Male specimens (in column) are represented in blue. Female specimens (in line) are represented in red.*

The symbol “*” refers to the sub-table represented in Table 1B.

*For each breeding, the respective progeny is represented within each box of the grid.*

WT [wild-type].

KO [homozygote knocked-out for the DAT gene] usually obtained as sibling of other epi-genotypes.

QULL [this category includes either “NULL” specimens, pups of both KO parents, or “QU” specimens where “U” stands for the “KO uterus,” homozygous offspring of a KO female and a HET male, belonging to the half pups that have inherited the truncated-DAT allele from the heterozygous father]. Since no behavioral or phenotypic differences were noted between the two epi-genotypes, it was chosen, for practical reasons, to unify them under the common abbreviation “QULL.” Each time we use QULL rather than KO, the offspring also has a KO grandmother.

MAT [heterozygous specimens, offspring of a KO male and a WT female, in which the wild allele comes from the maternal lineage, hence the abbreviation]. These offspring are likely to inherit from their father some unknown patterns of epigenetic modulations (Carbone et al., 2020; Zelli et al., 2021).

PAT [heterozygous specimens, which are the “specular” version of MATs but in which the wild allele comes from paternal lineage, hence the abbreviation]. They are, in fact, pups of a WT male and a KO female; therefore, the fetus develops in a likely hyperdopaminergic uterus. Also, they shall be fostered, at birth, to another dam. In fact, KO dams do not lactate; therefore, the offspring is found to die of starvation within 24 h of birth.

MIX by MAT [or simply “MIX,” heterozygous offspring of a MAT mother and a KO father, asset M]. It is important not to confuse them with those originally named as “MIX-HET,” and currently renamed HET^2^: classical offspring of both heterozygous parents for which it is impossible to track the onset of the hypofunctional DAT gene (see section “Sociality and Spontaneous Circadian Rhythms of Locomotor Activity in MAT-HETs”). They have been used as a line of comparison during the original research (Carbone et al., 2020; Zelli et al., 2021). In our next studies, we will permanently use the new code HET^2^ to refer to these specimens. Similarly, “MIX by PAT” ones, conventionally re-named “PIX,” are instead pups of a PAT mother and a KO father.

MUX [heterozygous offspring of a KO female and a MAT male, asset P]. As explained above, the offspring has a WT grandmother and a KO grandfather. Therefore, the initial letter “M” underlines the original maternal genealogy of the wild allele (coming from the paternal grandmother), while the letter “U” indicates fetal development in a “KO uterus” (hyperdopaminergic environment, in this case the body of a KO mother) plus the necessity of postnatal fostering, as well as for PAT rats. The consideration of hyperdopaminergic uterus in KO mothers opened us to the idea that KO rats may also be obtained in this way (see QULLs above: in this case, not only the subject is, of course, hyperdopaminergic, it also develops in such uterine environment).

μAT [“*micronAT*,” similar in characteristics to MAT but offspring of a QULL male instead of a KO one, and of a WT female]. Therefore, it was decided to use the Greek letter “*micron*” in order to maintain the name assonance and, at the same time, differentiate the worsened origin of the truncated-DAT allele: in this case coming from a KO father who is also developed, as a fetus, in a KO dam.

At this point, it was decided to realize a genealogical table keeping the criteria chosen to name each line of interest. The idea is to maintain the same basic-framework logic in order to also attribute, to all the specimens obtained from the different combinations of crossing, equally coherent nominative abbreviations. Therefore, following the table, for ease of reading and interpretation, the chosen order is that which conventionally goes from left to right. Thus, we find the following types of specimens:

### When the Dam Is a WT

WT × WT: WT [wild-type].

WT × KO: MAT [described above] (see section “Criteria and Genesis of Nominative Abbreviations”).

WT × QULL: μAT [described above] (see section “Criteria and Genesis of Nominative Abbreviations”).

WT × MAT: WAT [we opted for this specific abbreviation because it is of simple and intuitive understanding: it results from the fusion of the maternal (WT) and paternal (MAT) inputs; therefore, it keeps assonance with “MAT” even if, unlike the latter, it does not have a KO father and has WT siblings]. Notably, the father is a heterozygote: offspring has a paternal KO grandfather and both WT grandmothers; therefore, the putative epigenetic marks (on DAT-trunk allele transmitted by a KO male) are moved on the preceding generation (grandfather instead of father) compared to the MATs. The wild allele is inherited by maternal lineage (asset M), previously termed MAT-M (Oggiano et al., 2022).

WT × MIX: WIX [asset M: offspring of a WT female and a MIX (by MAT) heterozygous male, which in turn is offspring of a MAT female (and a KO male)]. Consistently with the logic followed in order to name the previous one, this abbreviation derives as well from the fusion of the parental inputs. The original DAT trunk allele is (compared to WAT) now moved to the great-grandfather and converged to this offspring by a paternal heterozygous grandmother.

WT × MUX: YOT [initially named WUX (asset M): criteria were the same as above; therefore, the acronym was chosen because it was derived from the fusion between WT (the mother) and MUX (the father)]. Later, though, we decided to rename it. In fact, since the WT mother does not carry the altered gene at all, the second letter “U” would be inappropriate. The initial “Y” was chosen, since the father is offspring of a KO female and a MAT male; therefore, he developed within a hyperdopaminergic uterus, and its own sperm may perhaps transmit sequelae of that. The same use of letter “Y” can be found for the MYX pups. Lastly, letters “OT” allow us to immediately recall that the healthy allele is inherited both from the mother and maternal grandmother.

### When the Dam Is a KO

KO × WT: PAT [described above] (see section “Criteria and Genesis of Nominative Abbreviations”).

KO × KO: NULL [part of QULL] (see section “Criteria and Genesis of Nominative Abbreviations”).

KO × QULL: [part of QULL] (see section “Criteria and Genesis of Nominative Abbreviations”).

KO × MAT: MUX [described above] (see section “Criteria and Genesis of Nominative Abbreviations”).

KO × MUX: YUX [the mother is KO for the DAT allele, while the father is a heterozygous offspring of a KO female and a MAT male (asset P)]. The acronym for this heterozygote was chosen with the intent of maintaining coherence with the second letter “U” denoting birth from KO mother (i.e., development in hyperdopaminergic uterus), indicating that the sperm was from a father that developed in a “KO uterus” as well. As such, here we have both direct development under hyperdopaminergia and sequelae of this carried by sperm. The first letter “Y” reverses them from YOT simply by replacing a KO female with a WT dam (× MUX male: thus, we highlight the fact that, in this case, the only healthy allele derives from the paternal grandfather only denoting odd inheritance that is paternal).

KO × MIX: MUX^2^ [asset P by inversion of parents, compared to MIX^2^]. We decided to study the effects of the hyperdopaminergic uterus due to hypofunctional DAT gene also in a third generation, following maternal and paternal lineages. This consists of the offspring of a KO female and a MIX, instead of MAT, male, and therefore similar to MUX but on next generation, notably an exact opposite case compared to that of MIX^2^ [offspring of a MIX instead of MAT female and a KO male, see section “When the Dam Is a MIX (by MAT)”]. For convenience, it was decided to keep the same logic, where the letter “U” within the name MUX^2^ indicates provenience of pups from the “KO uterus” of dams.

### When the Dam Is a QULL

QULL × WT: πAT [the dam here is not simply KO but also developed in hyperdopaminergic uterus: it can indifferently be either “NULL” (of both KO parents) or “QU” (of a KO female and a HET male, which inherited the truncated DAT allele from the heterozygous father)]. In order to immediately identify the presence of the “Null” allele (a truncated DAT allele carrying putative additional epigenetic regulation because it developed within a KO uterus), we decided to add the Greek letter “π” (“*pi*”) and keep the remaining part of the name in strict assonance with PAT (whose mother instead is a simple KO): the Greek letter highlights the putative presence of worsened maternal inheritance.

QULL × KO: QULL [described above] (see section “Criteria and Genesis of Nominative Abbreviations”).

QULL × QULL: [not included because never born yet].

QULL × MAT: NUX [given the close relationship with MUX (whose mother is simply KO), it was decided to attribute a similar acronym, but in this case, the initial has been mutated to “N”]. The letter “N” is indicating, in fact, the “Null” rather than the “simply” KO allele, inherited from the mother that developed in a hyperdopaminergic uterus. The “U” letter denotes that the dam has a “KO uterus” herself, where this offspring develops in a hyperdopaminergic environment as well.

QULL × MIX: NUX^2^ [as for MUX^2^, the abbreviation was attributed for convenience to maintain coherent assonance and logic of reasoning, with the use of the letter “N” to denote the “Null” rather than simply KO allele of the latter].

QULL × MUX: YUX^2^ [similar to the case of YUX, this heterozygous offspring inherits the healthy allele exclusively from the paternal grandfather (the father, MUX, is the son of a MAT father, proposing again only paternal inheritance of healthy allele). However, unlike YUX, the mother here is a QULL]. For this reason, a similar nominative acronym has been maintained but with the addition of the “square” symbol (which would recall a double “U”) precisely emphasizing that the pups developed themselves within a “KO uterus” genealogically descending from a hyperdopaminergic uterus, on top of sperm conveying the healthy allele but carrying a “KO uterus” mark (initial letter “Y”).

### When the Dam Is a MAT

MAT × WT: SOT [corresponding to MIX-P in Oggiano et al. (2022); it was originally named MAW, which was derived from the fusion of maternal and paternal abbreviations. As explained earlier, asset P is “sperm-originated”; therefore, we decided to change it to SOT, an abbreviation that seemed more appropriate to represent this concept]. In order to maintain vague assonance with the MAT and MIX abbreviation, we have also named SIX the progenies of MIX mothers [see section “When the Dam Is a MIX (by MAT)”] plus WT fathers.

MAT × KO: MIX (by MAT) [not to be confused with MIX^2^, offspring of both heterozygous parents]. Asset M by inverting the genotype of the father from WT to KO compared to SOT.

MAT × QULL: NIX [the “N” letter denotes a “Null” allele, since the KO father also developed in a hyperdopaminergic uterus. It represents the opposite case with respect to NUX (both pups of a QULL and a MAT parent)]. Therefore, in both the MIX and NIX cases, with the mother being a MAT, it was decided to leave the initial and keep the remaining unchanged and assonant).

MAT × MAT: [GDIX but is not included because not significant] (see section “Some Further Rules for HET × HET Couples”).

### When the Dam Is a MIX (by MAT)

MIX × KO: MIX^2^ [asset M: at a point, KO males were mated with MIX females, descending from MAT, in turn offspring of the initial WT females (Pepe et al., in press): like, for MYX, we decided to study the effects of the hypofunctional DAT gene on a third generation]. To follow the distribution of the allele by maternal lineage, always with KO fathers, we obtained a 3rd-generation heterozygous offspring named MIX by MIX; hence, for simplicity, the name was shortened to MIX^2^.

MIX × WT: SIX [asset P: by inverting paternal genotype to WT, compared to MIX^2^; the acronym has been selected in concordance with all pups of MAT dams, such as MIX by MAT (MAT female × KO male), NIX (MAT female × QULL male), and GIX (MAT female × PAT male)]. For this reason, the final “IX” was maintained. Through this nominative acronym, our intention is to underline the origin of the wild allele (which is “sperm-originated” like for SOT). For this case, we crossed MIX (by MAT) females and not MAT ones (which in fact were their mothers). Also, where the initial letter “S” has been added, wild allele for MIX^2^ and for SIX comes from HET maternal grandmothers, whereby for MIX-by-MAT and SOT it comes from WT maternal grandmothers.

MIX × QULL: NIX^2^ [again, like MIX^2^ is offspring of a KO male, this one is offspring of a QULL male (carrying the “Null” allele): it represents the exact opposite case of the NUX^2^, parents being inverted]. The abbreviation was attributed for convenience in order to maintain coherent assonance and logic of reasoning, with others (MIX^2^, NUX^2^).

MIX × MIX: MIIX [special case, described later] (see section “Some Further Rules for HET × HET Couples” and Table 1B).

**TABLE 1B:** Acronyms for HET × HET breedings.

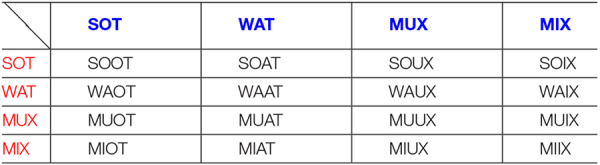

*Male specimens (in column) are represented in blue. Female specimens (in line) are represented in red.*

*For each breeding, the respective progeny is represented within each box of the grid.*

MIX × MAT: [not included because not significant for the purposes of the current study].

MIX × MUX: MIUX [special case, described later] (see section “Some Further Rules for HET × HET Couples” and Table 1B).

### When the Dam Is a MUX

MUX × WT: YAW [asset P with the healthy allele that derives from the father: initially named SUX, because of assonance with SOT (MAT female × WT male) and SIX (MIX female × WT male), where the letter “S” denoted a “sperm-originated” wild allele]. Later, it was renamed YAW: we preferred this abbreviation to be consistent with YUX, YUX^2^, and YOT. Indeed, the second letter “U” would be inappropriate. Rather, the first letter “Y” seemed more appropriate to make the abbreviation indicate that the mother, MUX in this case, is offspring of a “KO uterus” and may convey putative epigenetic marks.

MUX × KO: MYX [to keep assonance, because from a MAT or MIX female and a KO male we obtain MIX or MIX^2^ offspring, the mid letter “Y” in this specific case remembers that the mother, MUX, is the heterozygous offspring of a KO female (and a MAT male). Maternal transmission conveys a “Null” allele with putative marks from the hyperdopaminergic uterus of a grand dam. Father is WT for YAW (asset P), while here the father is inverted to KO: the abbreviation was chosen with initial letter to recall the “M” origin of the healthy allele (deriving, of course, from the maternal grandfather)].

MUX × QULL: NYX [again, the abbreviation was chosen because it is in assonance with MYX (offspring of a MUX female and a KO male) but the father in this case is a QULL. Pups have asset M but their mother, MUX, is the heterozygous offspring of a KO female and a MAT male]. Therefore, the letter “N” denotes the “Null” allele.

MUX × MAT: [these specimens are named GDIX-φu for the following reasons: starting from a MAT × MAT breeding, whose offspring is GDIX, we may change the mother into a second-generation one; similarly, the case of MUX × μAT (σIX-φu pups) is a second-generation dam for PAT × μAT (ΔIX pups)]. Now, as detailed just above, we may use Latin letters when changing the simply-KO-allele-bearing lineage, and Greek letters when changing the NULL-allele-bearing lineage. Hence, the suffix “φu” was used.

MUX × MIX: MUIX [special case, described later] (see section “Some Further Rules for HET × HET Couples” and Table 1B).

MUX × MUX: MUUX [special case, described later] (see section “Some Further Rules for HET × HET Couples” and Table 1B).

### Less Frequent Couples

WT × PAT: POT [initially named P-WAT because it denotes a “WAT with PAT-instead-of-MAT father”; it was later renamed POT in order to have a simpler abbreviation and maintain assonance with SOT]. PAT fathers are heterozygous that developed in a hyperdopaminergic uterus just like MUX fathers; similar to YOT pups, in POT pups (asset M), the mutated allele comes from paternal lineage but may carry signs of inheritance through sperm generated in gonads that, in turn, developed under hyperdopaminergia (being their father’s offspring of a KO female). Initial letter “P” (similarly to initial “Y”) allows to underline this important characteristic while maintaining nominal consistency with the criteria of the other abbreviations.

WT × μAT: μOT [initially abbreviated as μ-WAT because of similarity with WATs (born from MAT male) but with the difference that here the father is a “*micronat”* and, therefore, carries the “Null,” and not simply KO, allele (inherited from its own QULL father and carrying putative marks of hyperdopaminergic uterus of the KO great-grandmother)]. Like for POT, the “WA” was merged into “O”; hence, it was later changed to μOT (“*micron*OT”). Once again, the logic used for the previous specimens has been consistently maintained.

KO × PAT: PUX [the criterion used for this specific abbreviation is very intuitive: the initial “P” merges the name of the PAT father denoting the paternal origin of the wild allele; “U” underlines birth from a “KO uterus” making an offspring with a double uterine hit, directly of a KO mother and indirectly of a father that is son of KO paternal grandmother]. The name, as a whole, maintains global assonance with MUX (offspring in the paternal line of a KO grandfather and WT grandmother due to MAT rather than a PAT father). Passing from MUX to PUX means inverting the grandparent hit from sperm to uterus.

KO × μAT: μUX [following the logic explained above, the letter “*micron*” indicates the nature of the father (i.e., by QULL grandfather who developed in a hyperdopaminergic uterus of KO great-grandmother) and, therefore, the presence during spermatogenesis of the “Null” allele; the letter “U” denotes their direct origin from a “KO uterus”]. Note that μUX received the wild allele from fathers (asset P). The question here is whether such a wild allele takes marks from interaction with the “Null” allele compared with MUX pups. The entire abbreviation keeps assonance with the previous ones.

QULL × PAT: πUX [inversion of PAT × QULL; asset, therefore, is P, where the mother carries the “Null” allele, while the father is son of a KO female and may carry marks of her uterus]. Thus, initial Greek letter “π” (“*pi*”) follows the logic according to which it denotes, compared to PUX, the presence of the “Null” allele. Second letter “U” is used because pups developed directly in a “KO uterus”. Both wild and truncated-DAT alleles carry putative additional epigenetic regulation, related to hyperdopaminergic uteri (the latter directly and the former indirectly from grandmother *via* the paternal sperm). Also, the remaining part of the name is in strict assonance with the previous ones.

QULL × μAT: νUX [these specimens have origin to similar that of μUX (whose mother, though, is KO) and to πUX and NUX (whose fathers are, respectively, PAT and MAT)]. It has a QULL mother rather than KO. In this case, it was decided to conveniently turn the initial into a Greek letter: we have hence chosen “ν” (“*ni*”) for clear assonance.

## Miscellaneous

The following are HET × HET crosses, whereby one partner is first-generation (MAT, PAT, and μAT); and the other partner are second-generation (MIX and MUX but could also be μIX or μUX), in these cases the mother. To be honest, originally, we planned to use second-generation fathers, but then we discovered that a crucial role may be played actually by mothers being first- or second-generation. It was chosen to adopt special suffixes such as “-f,” “-fu,” “-ϕ,” and “-ϕu,” where “f” (and “ϕ”) may stand for “following generation” (or even “first encounter sequelae over”) in order to emphasize that these subjects are offspring of dams of a subsequent generation. Despite having a vulnerable HET epigenotype, they may be “protected” by grandmaternal transgenerational inheritance. In fact, epigenetic regulations seem to exert their influence through transgenerational effects. According to what was explained in one our recent study (Pepe et al., in press), when the mother is MAT (daughter of a WT female and a KO male), the “wild” and “mutated” alleles meet for the very first time, thus causing a series of epigenetic anomalies in the progeny. By replacing a MAT with a MIX female, therefore with a second-generation dam (since a MIX is daughter of a MAT mother and a KO father), effects on pups appear rather mitigated.

### Various HET × HET Couples

MAT × MIX: [not included] (see section “Less Frequent Couples”).

MAT × MUX: [not included] (see section “Less Frequent Couples”).

MAT × PAT: GIX [abbreviation for “Grandmother-biased” or “grandmaternal heterozygous”]. MATs are daughters of a KO male and a WT female, in which the wild allele comes from the maternal lineage; PATs are sons of a WT male and a KO female, and their wild allele comes, therefore, from paternal lineage. In GIX, referring to 2/4 of heterozygous offspring, the functional DAT allele is inherited from the maternal grandmother or paternal grandfather (asset MM or P). Hence the name, which is decided based on (grand) maternal lineage.

MAT × μAT: σIX [the Greek letter “σ” indicates the “*micronat*” nature of the father and, therefore, the presence in half of the offspring of the “Null” allele coming from the hyperdopaminergic uterus where the paternal grandfather developed]. This specific letter has been chosen to maintain assonance with the σOTs, putative offspring of a μAT female.

MIX × PAT: GIX-f [as described above, the abbreviation GIX (“Grandmother-biased heterozygous”) allows for the identification of the grandmaternal derivation of the wild functional DAT allele (the maternal grandmother in GIX). The specific case here is that of breeding a MIX rather than a MAT female with a PAT male. Therefore, here, it was decided to adopt the same basic GIX abbreviation, although an identifying suffix was added].

MUX × PAT: GIX-fu [similarly as above, the mother is a second-generation offspring while the father is a first-generation one]. We followed exactly the same logic described above. In this case, though, the nominal suffix is “-fu,” where “u” underlines the origin of mother from “KO uterus,” since MUX is born from a KO female (grandmothers of the offspring are both KO).

MIX × μAT: σIX- ϕ [also in this case, the reasoning for attribution of the abbreviation is consistent with what has been explained above, though, the father is a μAT (son of WT female × QULL male)]. Therefore, the “Null” allele is passed on through subsequent generations by the paternal grandfather like already seen for σIX. Based on what was previously explained for similar cases, Greek letters are used in the nominative abbreviation.

MUX × μAT: σIX- ϕu [like the previous case, the suffix also contains the letter “u” to underline the presence of “KO uterus” in the genealogy (maternal grandmother is a KO). The father is a μAT (son of WT female × QULL male), the “Null” allele is, thus, passed on through subsequent generations by the paternal grandfather like already seen for σIX. However, together with the use of Greek letters, we may further propose for all the last cases to use “-f” and “-fu” to denote MIX or MUX dams, and “-ϕ” and “-ϕu” to denote μIX or μUX ones.

### Birth Lineage Cross-Breeding

The couples illustrated in this paragraph have a PAT dam, namely, a first-generation HET like a MAT but born from a KO dam. The mutated allele now comes to pups from the maternal grand-dam and no longer from the grand-dad. Preliminary observations on maternal behavior denote that PAT dams do not differ from a WT dam when lactating a HET litter. Hence, the crucial factor seems to be the mutated allele being transmitted by the male grandparent and not by the uterus of the female grandmother.

PAT × WT: PAW [this abbreviation is simple and of intuitive understanding: it is the fusion between the maternal (PAT) and paternal (WT) inputs]. Asset is P like for SOT, but here, it is fully P, along two generations. The mother is a heterozygote with hyperdopaminergic uterine development being offspring of a KO female. The PAW offspring carries the wild allele by paternal lineage with a WT male as father plus WT grandparents. However, compared to SOT themselves, dams similarly convey the mutated allele but this now comes from grand-dams, no longer from grandfathers (like for SOT). Here, we propose that the mutated allele from grandfather vs. grandmother may entail a decisive difference. Therefore, we made the choice to break the SOT vs. PAW assonance.

PAT × KO: PIX [originally they were named MIX by PAT, because they were heterozygous offspring of a PAT. Parallel pups of a MAT mother (and KO father) were already named “MIX (by MAT)”]. Later, we decided to conventionally rename them “PIX” in order to avoid confusion and to immediately be able to deduce the first paternal then maternal genealogy of the wild allele (inherited from the WT maternal grandfather).

PAT × QULL: πIX [asset M: the mother PAT is the daughter of a KO female and a WT male; the father also developed as a fetus in a hyperdopaminergic uterus: he can transmit the “Null” allele, rather than simply KO, to the offspring]. In order to maintain the assonance with PIX but at the same time highlight the difference between the two, consisting of the Null vs. KO mutated allele, the Greek letter π (“*pi*”) was chosen as the initial for the abbreviation.

PAT × MAT: DIX [this is the abbreviation for “granDad-biased heterozygous,” symmetrical to what has been explained previously for the GIXs]. PATs are daughters of a KO female and a WT male, in which the wild allele comes from the paternal lineage. MATs are sons of a WT female and a KO male; therefore, their wild allele comes from maternal lineage. Among the DIX pups the functional DAT allele is inherited from the paternal grandmother or maternal grandfather, assets PM or P, hence the name.

PAT × MIX: DIX-f [this is the opposite breeding with respect to MIX × PAT (in which offspring were named GIX-f)]. As already explained, offspring of all HET × HET breeding with MAT father and PAT mother have been named DIX (“granDad-biased heterozygous”) in order to identify the derivation of the wild functional DAT allele from the maternal grandfather or the paternal grandmother. Here, however, the latter will be heterozygous rather than wild-type simply by changing father from first- to second- generation. Therefore, it was decided to adopt the same basic abbreviation, although by adding the identifying suffix following the same reasoning used for similar cases previously described (“-f” if the father is MIX, and “-fu” if it is MUX).

PAT × MUX: DIX-fu [the logic is the same as the one applied for GIX-f, GIX-fu, and DIX-f above. The suffix “-fu” is used in order to underline the origin of the second-generation (“f”) father from a KO uterus (“u”). In this case, both the maternal and paternal grandmothers are KO].

PAT × PAT: [not included because not significant] BIX (see section “Some Further Rules for HET × HET Couples”).

PAT × μAT: ΔIX [consistent with the logic already followed in the case of DIX and σIX: the Greek letter “Δ” indicates the DIX with “*micronat*” nature of the father. Therefore, we underline the presence (in half of the offspring) of the “Null” allele (coming from a hyperdopaminergic uterus) that was not present in the lineage of DIX].

### When the Dam Is a μAT

μAT × WT: μAW [acronym derived from the fusion between maternal and paternal abbreviations, similar to PAW and MAW (even though the latter was later re-named SOT)]. The Greek letter is useful in order to immediately understand that a “Null” rather than a simply KO allele is inherited from the mother (asset P like for SOT) because of the QULL maternal grandfather, which developed in a hyperdopaminergic uterus.

μAT × KO: μIX [asset M: this breeding has similarities with that of MIX by MAT offspring made between a MAT female and a KO male, except that the mother in this case is a “*micronat*,” thus carrying a “Null” rather than a simply KO allele of grandpaternal lineage]. The offspring have a QULL grandfather: we maintained the full assonance specifying, however, the presence of the “Null” allele by means of the initial Greek letter “μ.” Even though a WT maternal allele is eventually transmitted, we need yet to verify if encounter of a NULL truncated-DAT allele, during gametogenesis carried by the mother, leads to epigenetic sequelae.

μAT × QULL: νIX [again, this breeding has similarities with that of MIX by MAT offspring made between a MAT female and a KO male, except for a double hit: the father is a QULL (instead of a KO, thus offspring has a KO paternal grandmother)] and the mother is a “*micronat*” (which has a QULL father herself)]. The sperm-transmitted mutated allele is “Null,” and the wild allele met a “Null” one during oogenesis. Such abbreviation maintains assonance with MIX and NIX: in this specific case, the initial Greek letter “ν” (“*ni*”) allows to underline the double presence of a “Null” allele: one is inherited from the father while the other had interacted with the wild one eventually transmitted from the mother.

μAT × MAT: θIX [despite the assonance with DIXs (whose mother is a PAT), these are also related to GIX and ΓIX but their KO maternal grandfather is replaced by a QULL maternal grandfather: it was decided to name these offspring using the Greek letter “θ” (“*Theta”*) whereby “TH” of *theta* stands for “Totally Healthy” maternal lineages]. We highlight the presence of the “Null” allele coming from the hyperdopaminergic uterus of great-grand dam, hence coming *via* QULL grandfather here. Of course, only half of these pups inherit the “Null” allele from the μAT female (which inherited it from her own QULL father) and have the wild allele from the father.

μAT × MIX: θIX-φ [this breeding shares some similarities with that just above (from which θIX offspring are obtained), although in this case we have a second-generation father with a μAT mother (carrying the “Null” allele inherited from her own father like all θIX)]. In order to underline the important characteristics, and as already explained, Greek letters have been used in the abbreviation (initial letter “θ” and suffix “-φ”). “θ” is an indication of the presence of the “Null” allele (in half of the offspring) from maternal grand dad, while “φ” underlines that the paternal allele derives from a HET grand-dam, not a WT one, serving second-generation fathers.

μAT × MUX: θIX-φu [the logic is the same as above. The suffix “-φu” allows for immediate evidence that there is a hyperdopaminergic uterus in the genealogy]. For this case, further epigenetic sequelae are to be attributed *via* the MUX father to the KO paternal grandmother. Grand-dam moves from WT to HET to KO as far as suffixes move from none to “-φ” to “-φu.”

μAT × PAT: ΓIX [this breeding has resemblance with that of GIX offspring. However, there is the presence of the “Null” allele (carried in all pups: conveyed to half of the pups by the μAT mother indirectly *via* the QULL maternal grandfather and to the other half of the pups by the PAT father directly from the KO paternal grandmother). Therefore the “Γ” (“*gamma*”) Greek letter has been used for assonance with letter “G”]. In ΓIX, as well as in GIX, maternal grandmother is WT, while maternal grandfather is a QULL or KO respectively.

μAT × μAT: [not included because not significant].

## Some Further Rules for HET × HET Couples

When classical HET × HET breeding is realized, there is no way to track parental origins, since the heterozygous offspring will be half with asset M and the other half with asset P. However, if we use HETs from our “biased” couples, some information is spared. For example, we may have PAT × MAT or μAT × MAT couples, or PAT × μAT ones, and still, even though the heterozygous offspring will be a mix of both assets, we keep trace of some biases, as described in sections “Criteria and Genesis of Nominative Abbreviations” and “Miscellaneous.” For comparison, pups born from MAT females have one paternal grand-dam, which is a KO or even QULL, while the maternal grand-dam is a WT (as is typical only of GIX and σIX, not of DIX or θIX pups). A special case is when the couple is MAT × MAT: GDIX, as the offspring has both KO grandfathers; therefore, the assets are really “mixed up.” In such a case, we can propose to keep one MAT parent fixed, preferably the mother, and decompose the inheritance by the father as if it was a WT male to transmit the wild allele, and a KO male for the DAT trunk allele. Just as an example, if we decompose GIX pups, we keep the MAT dam but split the male; if we decompose DIX pups, we keep the PAT dam. The combination of GIX subjects are the following: MIX (pups of MAT female and KO male, asset M) plus SOT (MAT female and WT male, asset P) plus their KO and WT siblings. For DIX case, decomposed subjects are PIX (KO male and PAT female, asset M), plus PAW (WT male and PAT female, asset P), plus their KO and WT siblings. Breeding that originates GIX pups can be brought to second-generation dam; accordingly, we would have GIX-f pups. When decomposing the father, we would have MIX × WT (SIX) and MIX × KO (MIX^2^) pups; these correspond accordingly to the SOT and MIX components, but with a second-generation dam. Specularly, breeding that originates DIX pups can be brought to second-generation dam, and accordingly we would have GDIX-φu pups. When decomposing the father, we would have MUX × WT (YAW) and MUX × KO (MYX) pups; these correspond accordingly to the PAW and PIX components but with a second-generation dam.

Lastly, we can propose to breed among them all second-generation HETs (see Table 1B). In essence, the idea is to breed together MIX and MUX, and WATs and SOTs, merging all pups of MAT parents. We have WT and KO grandmother or grandfather. Therefore, by one parent, the wild or KO allele is transmitted from the grandmaternal line, while by the other parent, the WT or the mutated one is transmitted through the paternal line. Here are two examples:

-MUX × WAT: MUAT [combined specimen, heterozygous offspring of MUX pups (KO female × MAT male) and WAT pups (WT female × MAT male). Its abbreviation derives from the fusion of the parental ones]. The HET grand-father is preserved, while grand-dams are radicalized: one lineage has a WT grand dam, and the letter “U” indicates grandmaternal “KO uterus”.

-MIX × SOT: MIOT [combined specimen, heterozygous offspring of MIX pups (MAT mother and KO father) plus SOT pups (MAT mother and WT father). Its abbreviation is the combination of the parental ones]. Here, the MAT grandmother is preserved; the genetic inheritance of the wild or the trunk allele is grandpaternally radicalized.

Finally, we introduced one last acronym (not included in Tables 1A, B because of hypothetical use):

BIX: [both grandmothers are KO, and both parents grew in a hyperdopaminergic uterus]. This could be the case for PAT × PAT and MUX × MUX couples.

## Conclusion

When working on a large number of epi-genotypes distributed over several generations, it is often easy to get confused. This, of course, can lead to both interpretative and practical errors. Today, computer software can be especially designed for organizing data analysis. However, during practical laboratory work, there is not always the time, or the opportunity, to make use of this software, as sometimes quick decisions are required. The need of a tool for immediate use prompted us to create a simple genealogical table of Mendelian inspiration. The usefulness of this system includes the optimized use of nominal abbreviations for identification of different lines of specimens, as well as ease of keeping track of all involved breeding.

Our studies are focusing in particular on analysis and evaluation in heterozygous rats, of the epi-genotypic consequences caused by the hypofunctional DAT allele within different generations. Therefore, it was first of all necessary for us to immediately identify the maternal or paternal inheritance of this allele throughout the different lines. This was obtained by adding the initial “M” (maternal) or “P” (paternal), indicating the origin of the wild allele to the nominal identifier of each progeny. Second, it was necessary to track each subsequent couple of parents in terms of difference from the initial one (our prototypic breeding is a WT with a KO, giving all heterozygous offspring). We have been replacing either the KO or the WT parent with a HET, also producing, of course, WT or KO siblings in the offspring, respectively. If the couple is WT × HET, we change the initial from “M” to “W” (upside down M) or initial “P” to “S” (for “sperm”), still tracking the asset. If the couple is HET × KO, we keep the initial to denote how the HET dam was generated (M,W,P, and S) and turn the “AT” into “IX” (reminiscent of “mixed,” denoting the presence in the litter of KO siblings). Along with this, it also became necessary to find a system to identify the “Null” rather than the simply KO allele by derivation from a “KO uterus” (namely from a DAT-KO female progenitor). This was done because behavioral sequelae in females with a “hyperdopaminergic” uterus are largely unknown (Henschen et al., 2013; Brancato et al., 2021). In this regard, it was decided to use the letter “U” or “Y” in the nominal abbreviations of the line with this characteristic (other than PAT specimens). The couple and asset of pups is inverted (e.g., in MUX, PUX, and μUX if compared to MIX, PIX, and μIX). The consequence is the following: while MIX pups have “normally” KO siblings, MUX pups have another type of KO siblings, which also developed in a hyperdopaminergic uterus; hence, the acronym becomes KU, or, better, QU. We finally termed “NULL” the offspring of both KO parents. Since no behavioral or phenotypic differences were noted between the two types, it was chosen, for practical reasons, to unify them under the common abbreviation “QULL.” QULLs are either “NULL” specimens (pups of both KO parents) or “QU” specimens, offspring of a KO female and a HET male: this progeny not only inherited two truncated-DAT alleles but also the passage through the uterus of the KO mother could well add further epigenetic modifications.

Finally, it was also important to be able to identify the presence, in the genealogy, of a “QULL” ancestor. For this purpose, we chose to use the initial letters of the Greek alphabet, like “μ” or “ν” recalling the “M” or “N,” thus having immediate access to this specific information. For all these reasons, the creation of nominal identifiers as indicative as possible of the main characteristics of the line, using precise and logical criteria, proved to be an optimal strategy and did strengthen the genealogical table itself.

## Data Availability Statement

The original contributions presented in the study are included in the article/supplementary material, further inquiries can be directed to the corresponding author/s.

## Author Contributions

AL and BC wrote the first draft of the opinion article. WA supervised the final version of the manuscript. All authors contributed to the article and approved the submitted version.

## Conflict of Interest

The authors declare that the research was conducted in the absence of any commercial or financial relationships that could be construed as a potential conflict of interest. The handling editor is currently co-organizing a Research Topic with one of the authors WA.

## Publisher’s Note

All claims expressed in this article are solely those of the authors and do not necessarily represent those of their affiliated organizations, or those of the publisher, the editors and the reviewers. Any product that may be evaluated in this article, or claim that may be made by its manufacturer, is not guaranteed or endorsed by the publisher.
